# The Effect of Crystalline Waterproofing Admixtures on the Self-Healing and Permeability of Concrete

**DOI:** 10.3390/ma14081860

**Published:** 2021-04-09

**Authors:** Anita Gojević, Vilma Ducman, Ivanka Netinger Grubeša, Ana Baričević, Ivana Banjad Pečur

**Affiliations:** 1 Faculty of Civil Engineering and Architecture Osijek, University Josip Juraj Strossmayer of Osijek, Vladimir Prelog Street 3, 31000 Osijek, Croatia; anitagojevic@gmail.com; 2 Slovenian National Building and Civil Engineering Institute, Dimičeva 12, 1000 Ljubljana, Slovenia; vilma.ducman@zag.si; 3 Faculty of Civil Engineering, University of Zagreb, Andrija Kačić Miošić Street 26, 10000 Zagreb, Croatia; ana.baricevic@grad.unizg.hr (A.B.); ivana.banjad.pecur@grad.unizg.hr (I.B.P.)

**Keywords:** concrete, crystalline admixture, self-healing, permeability, compressive strength, SEM, EDS, XRD, FTIR

## Abstract

This paper investigates the effectiveness of a specific crystalline waterproofing admixture (CWA) in concrete as a function of a water–binder ratio. Four concrete mixes with and without CWA were prepared; two of them with a water–binder ratio of 0.45 and two of them with a water–binder ratio of 0.55. Water permeability and compressive strength were tested on hardened concrete specimens and self-healing of cracks over time was observed. Cement paste and CWA paste were prepared to clarify the results obtained on the concrete specimens. SEM and EDS and XRD and FTIR were performed on the hardened pastes to explain the mechanism of CWA working. The results show that the addition of CWA had no significant effect on the compressive strength of the concrete, but reduced the water penetration depth in the concrete, and the reduction was more effective for mixes with lower water–binder ratio. Regarding the self-healing effect, it can be concluded that the addition of CWA improves the crack healing in concrete, but the efficiency of self-healing is highly dependent on the initial crack width. The mechanisms involved in the reduction of water penetration depth and crack healing in concrete can be explained by different mechanisms; one is creation of the CSH gel from unreacted clinker grains, then formation carbonate, and additional mechanism is gel formation (highly expansive Mg-rich hydro-carbonate) from magnesium based additives. The presence of sodium silicate, which would transform into carbonate/bicarbonate, also cannot be excluded.

## 1. Introduction

Like all other materials, concrete structures have their own lifetime; harmful substances from the environment penetrate the concrete and react with its constituents, leading to deterioration and/or corrosion of the embedded steel, thus affecting its durability. The durability of concrete largely depends on the properties of its microstructure, such as porosity and pore size distribution, since gases (e.g., CO_2_ from the atmosphere) and liquids (e.g., water in which aggressive ions are dissolved) can penetrate into the material through the pores [1]. In most cases, the reactants responsible for concrete degradation penetrate the concrete matrix with the water. Since the durability of concrete is one of the most important properties, both from a financial and a sustainability point of view, the reduction or better still the minimization of water penetration should be a top priority. It is well known that a carefully designed concrete mix, properly manufactured and executed (delivered to site, cast, vibrated and cured) and with a low water–binder ratio, results in a final product with low permeability and increased durability [2,3]. A well-known and widely accepted method to reduce the permeability of concrete mixtures and thus increase their durability is the addition of supplementary cementitious materials (SCMs) to concrete mixtures [4,5]. Due to the pozzolanic activity and the filling effect, the use of SCMs can result in high performance concrete that has both improved mechanical properties and reduced permeability, leading to improved durability [6,7,8].

Currently, special admixtures have been developed to reduce water penetration into concrete. According to ACI 212.3 R-16 [2,9,10], admixtures designed to reduce water penetration into concrete are classified into two subcategories: Permeability-reducing admixture for non-hydrostatic conditions (PRAN), formerly referred to as a “moisture repellent admixture” where resistance to water under pressure is very limited and is not suitable for concrete exposed to water under pressure, and permeability-reducing admixture for hydrostatic conditions (PRAH)—or a “waterproofing material” that is sufficiently stable to resist water under pressure and is used in watertight structures for tanks, foundations, containments, etc. [2,4,11]. Crystalline waterproofing admixtures (CWA) belong to the group of PRAH products, they are composed of Portland cement, specially treated quartz sand and a mixture of “active chemicals”, although the chemical composition of the active chemicals is kept confidential by all manufacturers [12,13]. These active chemicals react with the water and cement particles in the concrete and increase the density of calcium silicate hydrates in the concrete and produce pore-blocking precipitates in the capillaries and microcracks [10]. As a result, the water penetration depth in concrete with incorporated CWA should be lower compared to the water penetration depth in reference concrete, which is confirmed by the authors in [10,14] but also disputed in [15]. As previously described, CWA also promotes self-healing of cracks [16,17,18,19,20]. The research presented in [18] implies that each CWA produces different products/crystals responsible for reducing water penetration and self-healing of concrete. Regarding self-healing, the width of cracks that can heal completely varies in the studies; the completely healed cracks in [16,17] are up to 0.1 mm wide, in [18] up to 0.3 mm wide, while in the studies [19,20] even cracks with a width up to 0.4 mm were healed. According to [21], these admixtures are more effective in mixes with a higher water–binder ratio than in mixes with a lower water–binder ratio because higher water content in the mix promotes self-healing. In terms of other properties, CWA has been reported to improve the resistance of concrete to freeze–thaw cycles [22], reduce chloride ion penetration [22], improve resistance to sulphate attack [23], and not significantly affect the compressive strength of concrete [15,23]. Water absorption test results differed significantly among studies; CWA reduced water absorption in [22], did not affect water absorption in [23] and negatively affected water absorption in [15].

Studies dealing with water penetration of concrete under pressure either use a lower water pressure value than prescribed in the standard EN 12390-8 [14,15,16,24] or use a test regime with time-varying water pressure [20]. So far, only the authors in [10] have applied the water pressure prescribed in EN 12390-8 [25], but on concrete specimens cured significantly longer (90 days) than prescribed in that standard (28 days). The reason for the longer curing time of the concrete is probably due to the manufacturer’s instructions, according to which concrete with the CWA admixture should be preconditioned prior to water penetration testing, thus ensuring sufficient time for the CWA crystals to grow. However, according to the European standard, water penetration testing under pressure is strictly prescribed by EN 12390-8 [25] and should be followed.

The aim of this paper is to determine the efficiency and mechanism of action of a particular CWA that has recently appeared on the European market. The effectiveness of the CWA was tested on two concrete mixes with different water–binder ratios by measuring the water penetration depth under pressure (and compressive strength) according to the European standard and by monitoring the self-healing ability of cracks. An attempt was made to explain the mechanism of action of the CWA by SEM, EDS, XRD and FTIR methods on hardened cement/CWA paste specimens.

## 2. Materials and Methods

The experimental part of the study aimed to determine the influence of the crystalline waterproofing admixture (CWA) on the water penetration under pressure and reduction of crack widths in concrete, and to explain the mechanism of the CWA effect. For this purpose, it was necessary to conduct the study at two levels: (a) concrete and (b) cement/CWA paste. To determine the contribution of CWA on concrete properties, four concrete mixes were prepared and tested. Considering that crystal growth occurs exclusively in cement paste, cement paste and CWA paste specimens were prepared and used to explain the effect of self-healing using the methods scanning electron microscope (SEM), energy dispersive X-ray spectroscopy (EDS), X-ray diffraction (XRD) and Fourier transform infrared spectroscopy (FTIR).

### 2.1. Cement and Crystalline Waterproofing Admixture

**Figure 1:**
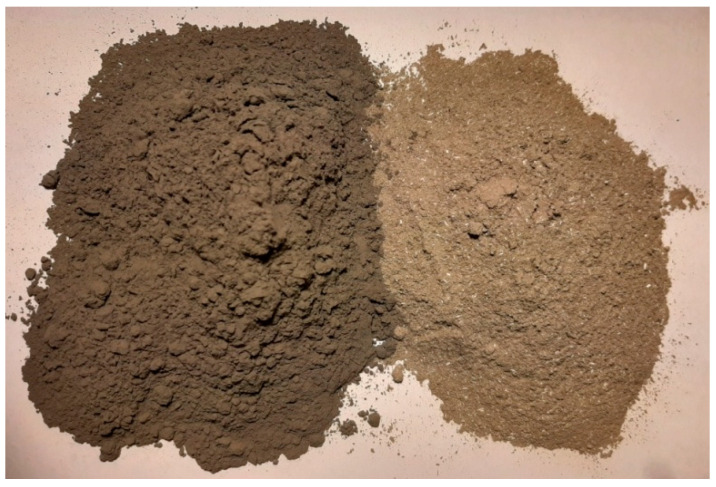
The appearance of cement (**left**) and CWA (**right**).

The cement used was Portland cement, CEM I 42.5 R, according to EN 197-1 [26]. The chemical composition of cement and CWA was determined by XRF (X-Ray Fluorescence) analysis using Thermo Scientific ARL Perform’X Sequential XRF (Thermo Fisher Scientific Inc., Walthem, MA, USA) on molten discs prepared by mixing sample and Fluxana (FX-X50-2, lithium tetraborate 50%/lithium metaborate 50%). The data listed in Table 1 were evaluated using the UniQuant program (Version 4.1). The density of the cement, measured according to EN 196-6 [27], was 3140 kg/m^3^. Figure 1 shows the appearance of cement (left) and CWA (right).

### 2.2. Aggregate

**Figure 2:**
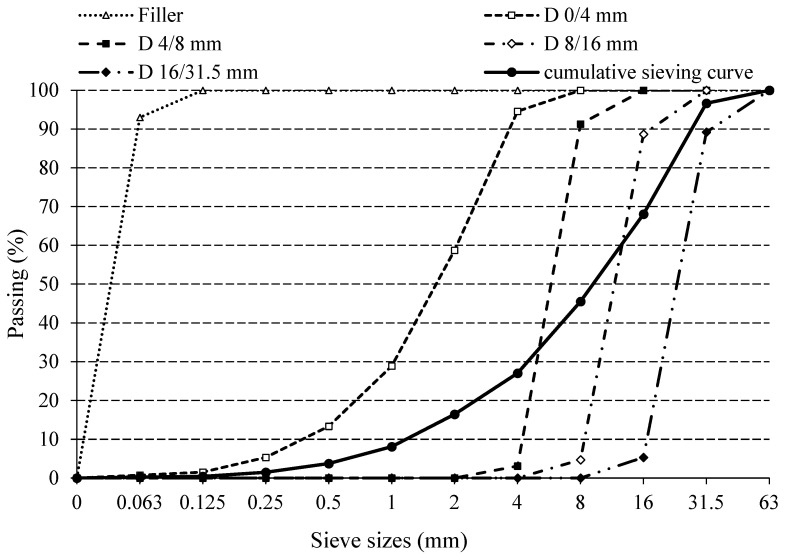
Particle size distribution of the aggregate.

All concrete mixes investigated in this study were prepared with dolomite aggregates (fractions corresponding to 0/4 mm, 4/8 mm, 8/16 mm and 16/31.5 mm) whose particle size distribution was determined according to EN 933-1 [28], as shown in Figure 2. The filler used was dolomite with passing of 93% for sieve size 0.063 mm and 100% for sieve size 0.125 mm. The density of the crushed dolomite aggregate was 2750 kg/m^3^, while the density of the filler was 2760 kg/m^3^ and tested according to EN 1097-6 [29].

### 2.3. Paste and Concrete Production

For the preparation of the paste mixtures, 500 g of binder (cement or CWA) and potable water were used. The water–binder ratio was 0.5. Four concrete mixes were prepared using cement, potable water, CWA and dolomite aggregate. Table 2 shows the proportions of the constituents in the mixes. All concrete mixes were prepared with a cement content of 350 kg/m^3^ and with potable water; two of the concrete mixes with a water–binder ratio (w/b) of 0.45 and two of them with a water–binder ratio of 0.55. The aim was to achieve two different consistency classes of fresh concrete mixes, S2 and S3 according to EN 206-1 [30]. In two concrete mixes, the CWA was added in an amount of 1% of the cement mass according to by the CWA manufacturer’s recommendation. In all concrete mixes, 5% of the mass of the 0/4 mm fraction was replaced by filler. Proposed designations for the concrete were: (a) M-0.45-R, (b) M-0.45-CWA, (c) M-0.55-R and (d) M-0.55-CWA, where the number represents the w/b ratio, R represents the reference mix and CWA represents the mix with added CWA.

The order of component mixing was as follows: 1. all aggregate fractions and filler were mixed in a pan mixer (DZ 100VS, Diemwerke, Hörbranz, Austria) for 2 min, then cement was added and mixed for 1 min and 2. water was added and mixed for 2 min. The crystalline waterproofing admixture (CWA) was added to the mixes containing it 1 min before the end of the entire mixing process, in such a way that it was evenly distributed in powder form over the mixing surface, where upon the mixing process was terminated.

The fresh state properties of the concrete were determined immediately after mixing. After casting, the concrete specimens were stored under cover for 24 h under laboratory conditions until demolding to prevent water evaporation. After demolding, the specimens were in the mist room at 20 ± 2 °C and RH ≥ 95% until the compressive strength test, i.e., after 28 days. Paste specimens with cement and with CWA were stored under the same laboratory conditions until the tests were performed (up to 2 months for SEM and XRD and up to 3 months for FTIR).

### 2.4. Methods

The microstructure of the cement paste samples was studied by scanning electron microscopy (SEM; Jeol JSM-5500LV, Jeol, Tokyo, Japan) under low vacuum conditions without coating. Chemical analysis of selected areas was determined by the SEM EDS method, supported by the Oxford INCA X-ray microanalysis system.

XRD analysis was carried out using Empyrean PANalytical X-Ray Diffractometer (Malvern PANalytical Empyrean, Malvern, UK) on unhydrated samples of cement and CWA, and on hardened cement paste samples after 56 days of hydration. Hardened cement paste samples were pulverized and scanned under clean room conditions from a 4 to 70° angle in 0.0263° increments.

Fourier transform infrared spectroscopy (FTIR) (Perkin Elmer Spectrum two, Hebron, KY, USA) of the unhydrated samples of cement and CWA and the paste samples after 95 days were recorded using a spectrometer (Spectrum Two, PerkinElmer, Waltham, MA, USA) in the reflection mode (Universal ATR) in the range 40–4000 cm^−1^ and with a resolution of 1 cm^−1^.

The concrete properties in the fresh and hardened state were tested according to the standardized procedures shown in Table 3. The concrete specimens for the self-healing tests were loaded to their compressive strength at 28 days of age. The crack width was measured on the cracked specimens before and after immersion in the water tank for 56 days. To observe the self-healing effect, 6 cracks per concrete specimen were randomly selected. A crack width ruler ranging from 0.10 to 2.50 mm with a step size of 0.05 mm and an illuminating magnifier with 6× magnification were used to observe the crack width.

## 3. Results and Discussion

### 3.1. Properties of Concrete

The test results of the fresh and hardened concrete properties are shown in Table 4. Each of the results presented here is an average of the three measurements. Comparison of the results in Table 4 shows that the M-0.45-CWA and M-0.55-CWA mixes achieved a compressive strength of about 97% compared to M-0.45-R and the M-0.55-R mix, indicating that the addition of CWA did not significantly affect the compressive strength of the concrete. The same conclusion is given in [15,23]. The slightly lower compressive strength of the mixes with CWA can be explained by the lower workability and higher air content of these mixes.

The water penetration depth of the M-0.45-CWA mix was 21% less than that of the M-0.45-R mix, while the water penetration depth of the M-0.55-CWA mix was 10% less than that of the M-0.55-R mix, leading us to conclude that the use of CWA reduced water penetration into the concrete. The reduction in water penetration depth obtained here by the addition of CWA is much lower than that obtained in [10], but higher than that obtained in [15], although the two studies mentioned here were conducted with a lower amount of CWA in concrete mixes. However, the concrete specimens tested in [10] were cured in a water tank for 90 days, while the concrete specimens in this study were cured for 28 days (as prescribed in EN 12390-8) under the same conditions. The curing time in the present study might be too short to ensure complete growth of CWA crystals. Possibly, a longer curing time or a higher amount of CWA could help to reduce the water penetration depth more efficiently.

According to the results presented here, CWA seemed to be more effective in mixes with a lower water–binder ratio, which is contrary to the conclusion given in [21]. Comparing the water penetration depths of the M-0.45-R and M-0.55-R mixes, it can be seen that water penetration depth can also be reduced at a lower water–binder ratio, as indicated in [2,3]. However, it should be noted here that the standard deviations for water penetration depth are significant, suggesting that the admixture was not uniformly distributed in the mixes.

### 3.2. Crack Width Reduction

**Figure 3:**
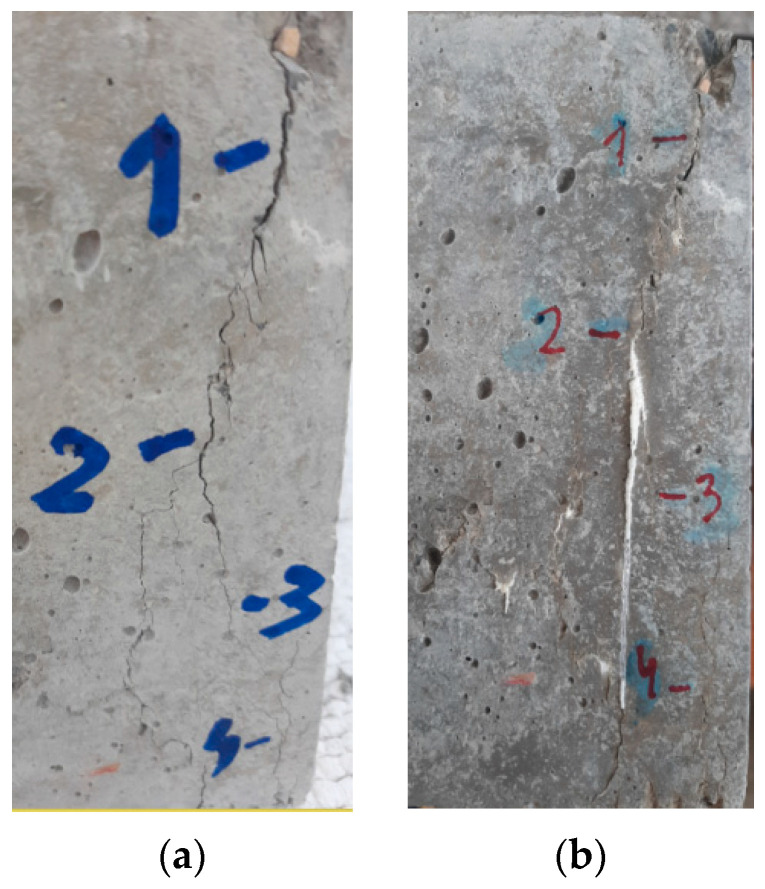
The appearance of cracks before (**a**) and after self-healing (**b**) in mixture M-0.55-CWA.

The occurrence of cracks before (a) and after self-healing (b) is shown in Figure 3 for the M-0.55-CWA mix. The results of cracking before and after curing of the concrete specimens in water are shown in Table S1 (Supplementary Materials). In order to estimate the degree of cracking in a particular concrete mix, the term initial cracking was introduced, which takes into account the number of cracks of a certain width that form immediately after loading. To evaluate the influence of CWA on the crack healing ability, the term crack reduction pattern was introduced, which observes the number of cracks of a certain degree of healing.

According to the authors in [35,36], in concrete with higher compressive strength, the initial cracks develop with a larger width than in concrete with lower compressive strength. From the observation of the initial crack pattern in Table S1, it cannot be concluded whether concrete with higher or lower compressive strength develops a larger number of cracks with larger width immediately after loading. Nevertheless, it can be observed (Table S1) that in the M-0.45-R mix, 6 cracks healed at a percentage greater than 90%, while in the M-0.45-CWA mix, 10 cracks healed at a percentage greater than 90%. In the M-0.55-R mix, 7 cracks healed while in the M-0.55-CWA mix, 10 cracks healed with the same percentage. Although it was concluded in [35] that the effect of self-healing is more pronounced in concrete with higher strength, this is not confirmed here—namely, the M-0.45-CWA and M-0.55-CWA mixes have the same number of cracks healed above 90%. It is known that cracks as small as 0.1 can be healed by autogenous healing [16,37]. However, considering the width of cracks healed over 90%, it can be observed that a larger number of cracks healed with a width of 0.15 mm in concrete mixes with CWA than in reference concrete mixes (3 cracks with a width of 0.15 mm healed in mixes M-0.45-CWA and M-0.55-CWA, while only 1 crack healed in mixes M-0.45-R and M-0.55-R). The influence of CWA on crack healing was thus confirmed. The fact that there are cracks up to 0.15 mm wide in mixes with CWA that did not heal completely indicates that the CWA is not evenly distributed in the mix. The individual results presented in Table S1 were further divided into three groups based on their self-healing ability, and an average crack width was determined for each mix and group. These results, shown in Table 5, indicate that CWA can ensure self-healing of cracks, but the efficiency of self-healing is highly dependent on the initial crack width.

### 3.3. Explanation of Self-Healing Mechanism

**Figure 4:**
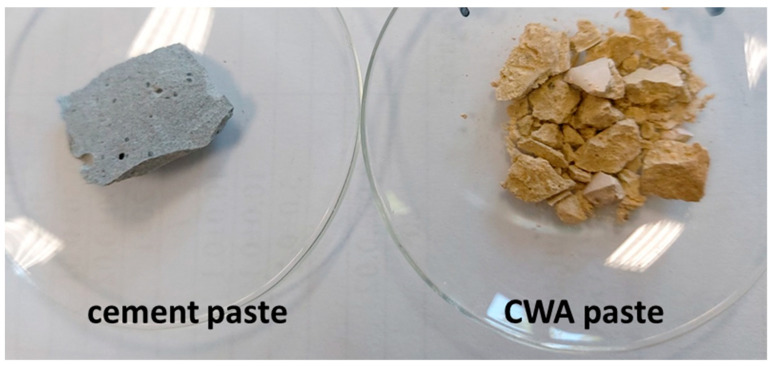
Macroscopic appearance of two pastes (cement paste and CWA paste).

To better understand the mechanisms of self-healing effects, pastes (instead of concrete) were prepared from cement (CEM) and CWA. The macroscopic appearance of the pastes, cement paste and CWA paste, is shown in Figure 4. Visually, CWA paste has a slightly yellowish color compared to the grey cement paste (Figure 4). CWA paste remains more “stickier” at 95% relative humidity and is more brittle than cement paste as it breaks easily under hand pressure.

**Figure 5:**
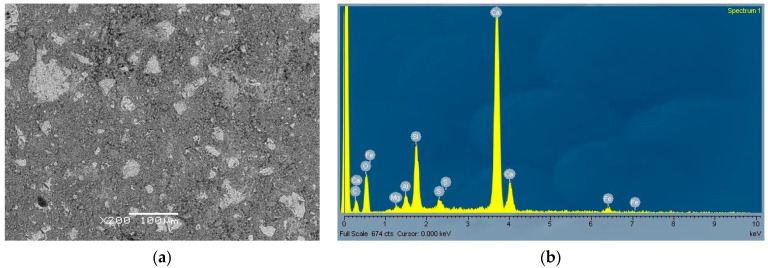
SEM image of cement paste—CEM (**a**) with corresponding EDS (**b**).

**Figure 6:**
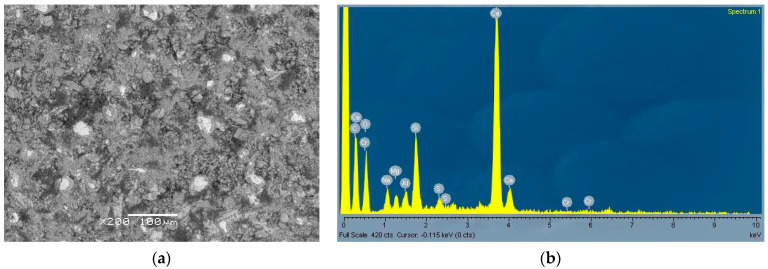
SEM image of CWA paste (**a**) with corresponding EDS (**b**).

**Figure 7:**
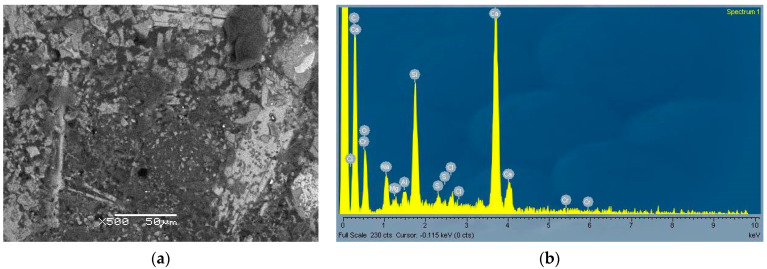
SEM image of CWA paste—darker area from Figure 6a (**a**) with corresponding EDS (**b**).

**Figure 8:**
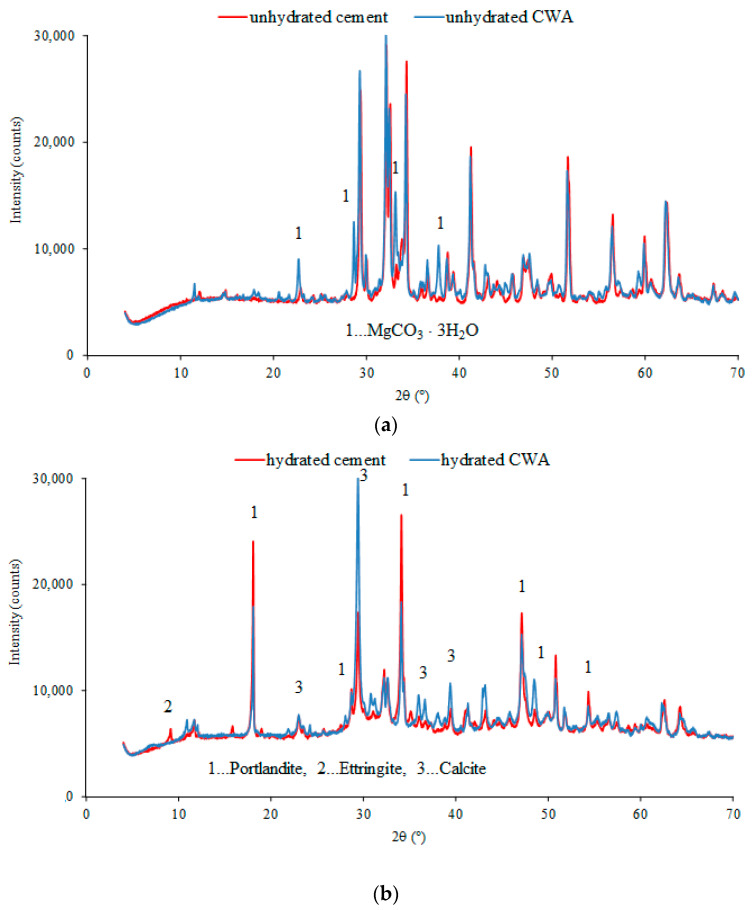
XRD diffractogram of cement and CWA: (**a**) before hydration and (**b**) after 57 days of hydration.

From Figure 5a and Figure 6a, typical microstructures of the two pastes can be seen with corresponding EDS analysis. While in the cement paste (Figure 5a) the microstructure was homogeneous and some unreacted clinker grains were present, in the CWA paste fewer (and smaller) unhydrated clinkers are observed and some inclusions (darker areas) are also seen. One of these areas is shown enlarged in Figure 7. The EDS analysis (Figure 6b and Figure 7b) confirms that CWA contained more sodium, carbon and magnesium compared to cement (CEM). In particular, the dark region (Figure 7a,b), which looks like an amorphous phase or gel, was enriched in sodium and carbon. The exact composition of CWA was not disclosed, but sodium and carbon could come from additives. Namely, it has been previously reported [38] that the addition of NaOH and Na_2_CO_3_ (optimum additive contents 1% and 1.5%, respectively) increases the strength of soil–cement mixtures. Similarly, the addition of water glass in a certain amount could help to increase the mechanical properties of concrete as it reacts with the calcium hydroxide to form calcium-silicate-hydrate (CSH) gel and in this way it can also heal cracks [39]. The chemical composition (Table 1) also confirmed that CWA contains much higher content of magnesium and especially sodium than cement. Since water glass can also be used as the main component to produce the concrete sealer, which provides good waterproofing properties [40]. If sodium silicate (water glass) is present in the system this would also contribute to increased amount of carbonate, as the sodium present in the mixture would react with CO_2_ from the air to form sodium carbonate or bicarbonate [41]. Additionally, a higher amount of carbonates was also detected in hydrated CWA paste as shown in Figure 8b, thus all these findings support the assumption that some powdered water glass (beside magnesium based components) was added into CWA. Since water glass had an amorphous structure and consequently could not be confirmed by XRD.

Following the research presented in [42], XRD analysis of the powdered material (cement and CWA) and hydrated pastes was performed to identify the additives and further clarify the mechanisms. In Figure 8a, the diffractograms show powders of unhydrated cement and CWA. Besides the expected phases in cements (C_2_S, C_3_S, etc.), the diffractogram of CWA contains additional peaks, marked 1 in Figure 8a, which could belong to magnesium carbonate trihydrate (nesquehonite). The complex MgCO_3_·3H_2_O nanostructures exhibit superhydrophobicity due to their unique superstructures, but the mineral can dissolve in NaCl solutions. The solubility of nesquehonite in pure water decreased with temperature, but in the presence of NaCl, the solubility of nesquehonite first increases to a maximum value and then gradually decreases with an increase in salt concentration [43,44]. Figure 8b shows diffractograms of both the cement paste and the CWA paste after hydration for 2 months, i.e., 57 days. As can be seen, there are some differences; in addition to the peaks belonging to cement hydration (calcite and portlandite), the appearance of new phase(s) in the diffractogram was noted (peaks at about 11° and 31°–32°), which need to be further identified. They could belong to the magnesium carbonate hydrate formed from nesquehonite when hydromagnezite is added to the CWA, but the amount is too small to be identified beyond doubt.

**Figure 9:**
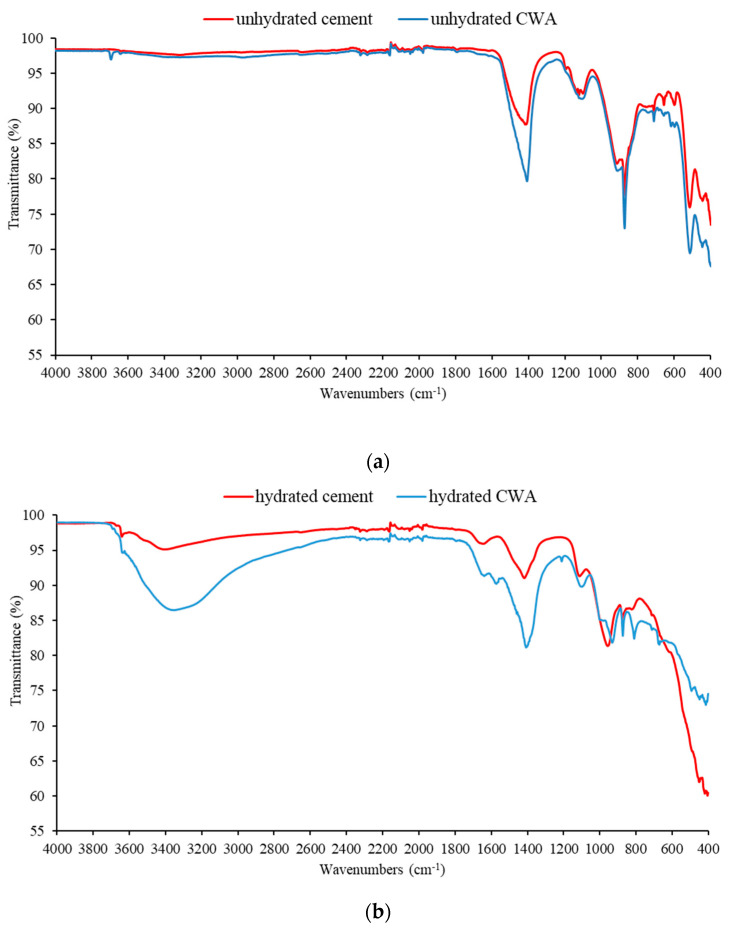
FTIR of cement and CWA: (**a**) before hydration and (**b**) after 95 days of hydration.

ATR-FTIR analyses were also performed on powdered material (cement and CWA) and on the hydrated samples at 95 days of age (Figure 9). All bands identified by FTIR (Figure 9a) are those commonly found in cements, and the only significant difference between CEM and CWA is a band at 3700 cm^−1^ attributed to Mg(OH)_2_ [45]. Some carbonates could also be present, e.g., a single asymmetric stretching band observed between 1450 and 1400 cm^−1^ could be attributed to MgCO_3_. After 95 days of hydration, there were two major differences between cement and CWA in the hydrated samples (Figure 9b); the broad band at about 3400 cm^−1^ corresponding to the O−H stretching vibrations (v1 and v3) in the water molecules was much more pronounced in CWA than in cement, confirming that CWA retains water [46]. The band in the range 1420–1480 cm^−1^ is attributed to CO_3_^2−^/HCO_3_^−^ [45], confirming the formation of more carbonates in CWA. These carbonates could fill the cracks or voids and consequently contribute to a denser structure and less water penetration.

Drawing a parallel with a similar study presented in [10], one could conclude that the mechanisms involved in this reduction of water penetration into the concrete matrix are related to different types of chemical reactions, as clearly explained in [22]; hygroscopic crystallization, hydrophilic crystallization and hydrophobic characteristics. Thus, firstly, the hydration of the unhydrated admixture particles leads to more calcium hydroxide gels when water penetrates into the concrete. In the case of CWA, more carbonate phase is also formed, which is confirmed by XRD. The Mg(OH)_2_ present in CWA could dissolve and recrystallize to nesquehonite upon water penetration into the concrete, according to the mechanisms described in [47]:

Mg(OH)_2_ + CO_2_ + H_2_O → MgCO_3_·3H_2_O (1)

Ca^2+^ + Mg^2+^ + CO_3_^2−^ → Ca_x_ Mg_(1−x)_ CO_3_ (2)

The reaction may continue to dypengite and hydromagnezite, both of which incorporate water into the structure. Such reactions that incorporate water into the structure could also be present in CWA. This is the reason why CWA has a much more pronounced band at about 3400 cm^−1^ than cement paste. This band corresponds to the O−H stretching vibrations in the water molecules, which, as seen in the FTIR (Figure 9b), is present in CWA and not in the CEM hydrated paste.

In summary, several mechanisms could be involved in the present system; one is the formation of CSH gel from unreacted clinker grains and subsequent carbonate formation, another mechanism is gel formation (highly expansive Mg-rich hydro-carbonate) from magnesium-based additives [47]. The presence of sodium silicate (based on the chemical analysis, where the amount of sodium oxide was significant, and based on SEM Figure 7), which would convert to carbonate/bicarbonate, also cannot be ruled out [39,40]. The incorporation of these newly formed CSH phases, gels and carbonates into the pores improves the impermeability.

## 4. Conclusions

In this paper, the effectiveness of crystalline waterproofing admixture (CWA) in concrete mixes was studied as a function of the water–binder ratio. For this purpose, water permeability and compressive strength were tested and self-healing of cracks over time was observed. The methods SEM and EDS and XRD and FTIR were used to explain the effect of self-healing. The following could be concluded:

- The addition of CWA had no significant effect on the compressive strength of the concrete.
- The addition of CWA reduced the water penetration depth in the concrete, which was more effective for mixes with lower water–binder ratio. The curing time of 28 days, as prescribed in EN 12390-8, may be too short to ensure complete growth of CWA crystals and reduce water penetration depth more effectively. If the curing time of 28 days was followed, a higher amount of CWA could reduce the water penetration depth more efficiently.
- The addition of CWA improved the crack healing in concrete. Namely, a larger number of cracks with a width of 0.15 mm were healed in concrete mixes with CWA than in reference concrete mixes. The fact that there were cracks with a width up to 0.15 mm in mixes with CWA that were not completely healed suggests that the CWA was not evenly distributed in the mix, which could be solved by adding a higher amount of CWA to the concrete mixes.
- The mechanisms involved in the reduction of water penetration depth and crack healing in concrete can be explained by different mechanisms; one is the formation of CSH gel from unreacted clinker grains, then the formation of carbonate, and another mechanism is the gel formation (highly expansive Mg-rich hydro-carbonate) from magnesium based additives. The presence of sodium silicate, which would convert to carbonate/bicarbonate, also cannot be excluded.

## Figures and Tables

**Table 1:** Chemical composition of cement and crystalline waterproofing admixture (CWA).

Constituent	Cement	CWA
Chemical composition (% of mass)		
SiO_2_	19.18	13.50
CaO	61.98	46.74
Al_2_O_3_	4.85	3.47
Fe_2_O_3_	2.79	1.58
MgO	1.82	3.70
MnO	0.24	0.09
Na_2_O	0.25	9.30
K_2_O	0.80	0.63
SO_3_	2.73	2.42
Loss on ignition	4.54	18.11

**Table 2:** Concrete mix design.

Constituent/Mixtures	M-0.45-R	M-0.45-CWA	M-0.55-R	M-0.55-CWA
Cement (kg/m^3^)	350	350	350	350
Water (L/m^3^)	157.5	157.5	192.5	192.5
Water-binder ratio	0.45	0.45	0.55	0.55
Crystalline waterproofing admixtures (kg)	-	3.5	-	3.5
Aggregate in total (kg/m^3^)	1969	1969	1872.7	1872.7
Filler (kg/m^3^)	27.6	27.6	26.2	26.2
Dolomite, 0/4 mm (kg/m^3^)	523.8	523.8	498.2	524.4
Dolomite, 4/8 mm (kg/m^3^)	354.4	354.4	337.1	337.1
Dolomite, 8/16 mm (kg/m^3^)	452.8	452.8	430.7	430.7
Dolomite, 16/31.5 mm (kg/m^3^)	610.4	610.4	580.5	580.5

**Table 3:** Test methods for fresh and hardened concrete properties.

Property	Standard	Dimensions of Specimens	Number of Specimens/Testings
Density	EN 12350-6 [31]	-	3 per mix
Air content	EN 12350-7 [32]	-	3 per mix
Consistency	EN 12350-2 [33]	-	3 per mix
Compressive strength	EN 12390-3 [34]	15 × 15 × 15 cm^3^	3 per mix
Depth of penetration of water under pressure	EN 12390-8 [25]	15 × 15 × 15 cm^3^	3 per mix
Self-healing effect	-	15 × 15 × 15 cm^3^	3 per mix

**Table 4:** Test results of fresh and hardened concrete properties.

Property/Mixtures	M-0.45-R	M-0.45-CWA	M-0.55-R	M-0.55-CWA
Density (kg/m^3^)	2450 ± 15	2440 ± 19	2420 ± 17	2450 ± 20
Air content (%)	1.9 ± 0.1	2.1 ± 0.2	1.1 ± 0.1	1.4 ± 0.1
Slump test (mm)	80 ± 6	50 ± 4	150 ± 8	110 ± 7
Compressive strength (N/mm^2^)	50.9 ± 1.4	49.3 ± 0.8	44.6 ± 1.8	43.5 ± 1.1
Penetration of water (mm)	24 ± 2	19 ± 4	30 ± 8	27 ± 4

**Table 5:** Relationship between the self-healing contribution and initial crack width.

Mix ID	Self-Healing Contribution Based on the Initial Crack Width (mm)		
	0–20%	20–90%	100%
M-0.45-R	0.271	0.238	0.108
M-0.55-R	0.350	0.194	0.107
M-0.45-CWA	0.325	0.250	0.115
M-0.55-CWA	0.300	0.286	0.115

## Data Availability

The data presented in this study are available on request from the corresponding author.

## Supplementary Materials

The following are available online at https://www.mdpi.com/article/10.3390/ma14081860/s1, Table S1: Crack width before and after self-healing.
