# Supplementary material for: The Effect of Crystalline Waterproofing Admixtures on the Self-Healing and Permeability of Concrete
Source: Materials (Basel). 2021 Apr 9;14(8):1860. doi: 10.3390/ma14081860 (PMC8069652; doi:10.3390/ma14081860)
Supplement: Supplementary file 1 [file materials-14-01860-s001.pdf]

materials

MDPI

Supplementary Materials

The Effect of Crystalline Waterproofing Admixtures on the Self-Healing and Permeability of Concrete

Firstname Lastname [1], Firstname
Anita Gojević [1], Vilma Ducman [2], Ivanka Netinger Grubeša [1,*], Ana Baričević [3] and Ivana Banjad Pečur [3]

[1] Faculty of Civil Engineering and Architecture Osijek, University Josip Juraj Strossmayer of Osijek, Vladimir Prelog Street 3, 31000 Osijek, Croatia; anitagojevic@gmail.com
[2] Slovenian National Building and Civil Engineering Institute, Dimičeva 12, 1000 Ljubljana, Slovenia; vilma.ducman@zag.si
[3] Faculty of Civil Engineering, University of Zagreb, Andrija Kačić Miošić Street 26, 10000 Zagreb, Croatia; ana.baricevic@grad.unizg.hr (A.B.); ivana.banjad.pecur@grad.unizg.hr (I.B.P.)
* Correspondence: nivanka@gfos.hr; Tel.: +38531540070

Table S1. Crack width before and after self-healing

Mix ID	Specimen number	Crack ID	Initial crack width [mm]	Initial crack pattern	Crack width after selfhealing [mm]	Reduction in crack width (%)	Crack reduction pattern
M-0.45-R	1	1	0.25	0.1 mm ≤ width <0.2 mm – 9 cracks<br>0.2 mm ≤ width <0.3 mm – 6 cracks<br>0.3 mm ≤ width <0.4 mm – 1 crack<br>0.4 mm ≤ width <0.5 mm – 0 crack<br>0.5 mm ≤ width– 2 cracks	0.2	20	0% ≤ width reduction <20 % - 7 cracks<br>20% ≤ width reduction <90 % - 5 cracks<br>90% ≤ width reduction <100 % - 6 cracks
		2	0.55		0.45	18	
		3	0.1		0.1	0	
		4	0.2		0.2	0	
		5	0.3		0.2	33	
		6	0.2		0.2	0	
	2	1	0.5		0.45	10	
		2	0.25		0.25	0	
		3	0.1		0	100	
		4	0.1		0	100	
		5	0.1		0.1	0	
		6	0.1		0	100	
	3	1	0.15		0.1	33	
		2	0.1		0	100	
		3	0.25		0.1	60	
		4	0.15		0	100	
		5	0.1		0	100	
		6	0.25		0.15	40	
M-0.45-CWA	1	1	0.1	0.1 mm ≤ width <0.2 mm – 10 cracks<br>0.2 mm ≤ width <0.3 mm – 4cracks<br>0.3 mm ≤ width<0.4 mm – 3 cracks<br>0.4 mm ≤ width <0.5 mm – 0crack<br>0.5 mm ≤ width – 1crack	0	100	0% ≤ width reduction <20 % - 4 cracks<br>20%≤ width reduction <90 % - 4 cracks<br>90%≤ width reduction <100 % - 10 cracks
		2	0.3		0.3	0	
		3	0.1		0	100	
		4	0.2		0.2	0	
		5	0.25		0.2	20	
		6	0.5		0.5	0	
	2	1	0.1		0	100	
		2	0.1		0	100	
		3	0.15		0	100	
		4	0.25		0.1	60	
		5	0.3		0.25	17	
		6	0.15		0	100	
	3	1	0.15		0	100	
		2	0.3		0.15	50	
		3	0.1		0	100	
		4	0.1		0	100	
		5	0.2		0.15	25	
		6	0.1		0	100	

M-0.55 - R	1	1	0.3	0.1 mm ≤ width<0.2 mm – 12 cracks<br>0.2 mm ≤ width <0.3 mm – 4cracks<br>0.3 mm ≤ width <0.4 mm – 1 crack<br>0.4 mm ≤ width <0.5 mm – 0crack<br>0.5 mm ≤width– 1crack	0.2	33	0% ≤ width reduction <20 % - 2 cracks<br>20% ≤ width reduction <90 % - 9 cracks<br>90% ≤ width reduction <100 % - 7 cracks
		2	0.6		0.5	17	
		3	0.1		0.1	0	
		4	0.15		0.1	33	
		5	0.2		0.1	50	
		6	0.15		0.1	33	
	2	1	0.1		0	100	
		2	0.2		0.15	25	
		3	0.1		0	100	
		4	0.1		0	100	
		5	0.1		0	100	
		6	0.15		0.1	33	
	3	1	0.2		0.1	50	
		2	0.1		0	100	
		3	0.25		0.2	20	
		4	0.15		0	100	
		5	0.15		0.1	33	
		6	0.1		0	100	
M-0.55-CWA	1	1	0.1	0.1 mm ≤ width <0.2 mm – 11 cracks<br>0.2 mm ≤ width <0.3 mm – 2cracks<br>0.3 mm ≤ width <0.4 mm – 4 cracks<br>0.4 mm ≤ width <0.5 mm – 1crack<br>0.5 mm >width– 0 crack	0	100	0% ≤ width reduction <20 % - 1 cracks<br>20% ≤ width reduction <90 % - 7 cracks<br>90% ≤ width reduction <100 % - 10 cracks
		2	0.3		0.15	50	
		3	0.35		0.2	43	
		4	0.45		0.35	22	
		5	0.15		0.1	33	
		6	0.3		0.25	17	
	2	1	0.15		0	100	
		2	0.15		0	100	
		3	0.1		0	100	
		4	0.15		0	100	
		5	0.25		0.15	40	
		6	0.1		0	100	
	3	1	0.1		0	100	
		2	0.3		0.1	67	
		3	0.1		0	100	
		4	0.2		0.15	25	
		5	0.1		0	100	
		6	0.1		0	100	

Citation: Gojević, A.; Ducman, V.; Netinger Grubeša, I.; Baričević, A.; Banjad Pečur, I. The Effect of Crystalline Waterproofing Admixtures on the Self-Healing and Permeability of Concrete. *Materials* **2021**, *14*, 1860. https://doi.org/10.3390/10.3390/ma14081860

Academic Editor: Angelo Marcello Tarantino

Received: 9 March 2021
Accepted: 7 April 2021
Published: 9 April 2021

Publisher's Note: MDPI stays neutral with regard to jurisdictional claims in published maps and institutional affiliations.

Copyright: © 2021 by the authors. Licensee MDPI, Basel, Switzerland. This article is an open access article distributed under the terms and conditions of the Creative Commons Attribution (CC BY) license (http://creativecommons.org/licenses/by/4.0/).
